# Supplementary material for: Developing a Vaccine to Block West Nile Virus Transmission: In Silico Studies, Molecular Characterization, Expression, and Blocking Activity of Culex pipiens mosGCTL-1
Source: Pathogens. 2021 Feb 17;10(2):218. doi: 10.3390/pathogens10020218 (PMC7921969; doi:10.3390/pathogens10020218)
Supplement: Supplementary file 1 [file pathogens-10-00218-s001.pdf]

S1. Details of the mosquito samples in control and test groups used in the present investigation, code of samples, days post infection (dpi), and their SQ mean (RT-qPCR).

Group	Code of sample	Days post infection	SQ mean
**Control Group**	0C1	0	38354.56103
**Control Group**	0C2	0	36304.80286
**Control Group**	0C3	0	39655.23563
**Control Group**	2C1	2	3455417.31184
**Control Group**	2C2	2	1449647.31532
**Control Group**	2C3	2	56007.02659
**Control Group**	2C4	2	426463.04486
**Control Group**	2C5	2	221548.53702
**Control Group**	2C6	2	99567.07723
**Control Group**	2C7	2	53564.23570
**Control Group**	2C8	2	328756.82160
**Control Group**	2C9	2	28293.16023
**Control Group**	2C10	2	2730.20507
**Control Group**	2C11	2	136808.85414
**Control Group**	2C12	2	13903.11240
**Control Group**	2C13	2	912512.14130
**Control Group**	2C14	2	46955.88075
**Control Group**	2C15	2	7502.26892
**Control Group**	2C16	2	2810903.64767
**Control Group**	2C17	2	93802.07319
**Control Group**	2C18	2	2272834.15775
**Control Group**	2C19	2	1892821.21078
**Control Group**	2C20	2	420147.54206
**Control Group**	5C1	5	100543.76658
**Control Group**	5C2	5	369336.95239
**Control Group**	5C3	5	14835728.50820
**Control Group**	5C4	5	10381212.77059
**Control Group**	5C5	5	4905883.65391
**Control Group**	5C6	5	591045.01008
**Control Group**	5C7	5	0.00000
**Control Group**	5C8	5	30369153.43895
**Control Group**	5C9	5	22839692.16584
**Control Group**	5C10	5	74902.66250
**Control Group**	5C11	5	3008074.38720
**Control Group**	5C12	5	511521.09156
**Control Group**	5C13	5	44242577.72549
**Control Group**	5C14	5	316538.68816
**Control Group**	5C15	5	691172.81411
**Control Group**	5C16	5	1890962.53575
**Control Group**	5C17	5	2389.23798

**Control Group**	5C18	5	12748871.15846
**Control Group**	5C19	5	24415075.92153
**Control Group**	5C20	5	0.00000
**Control Group**	7C1	7	0.00000
**Control Group**	7C2	7	1261913.06671
**Control Group**	7C3	7	0.00000
**Control Group**	7C4	7	11486.44049
**Control Group**	7C5	7	1800443.97350
**Control Group**	7C6	7	3701514.10552
**Control Group**	7C7	7	7362.99167
**Control Group**	7C8	7	1419818.03533
**Control Group**	7C9	7	2771365.90971
**Control Group**	7C10	7	158546.30457
**Control Group**	7C11	7	846.53545
**Control Group**	7C12	7	2877936.96363
**Control Group**	7C13	7	1301.48074
**Control Group**	7C14	7	17280.84196
**Control Group**	7C15	7	1305572.72218
**Control Group**	7C16	7	2117846.82322
**Control Group**	7C17	7	9296.31370
**Control Group**	7C18	7	1145335.55056
**Control Group**	7C19	7	0.00000
**Control Group**	7C20	7	0.00000
**Test Group**	0T1	0	67625.83368
**Test Group**	0T2	0	69841.57595
**Test Group**	0T3	0	127828.39850
**Test Group**	2T1	2	71075.42184
**Test Group**	2T2	2	6932538.31093
**Test Group**	2T3	2	181627.72599
**Test Group**	2T4	2	190533.01349
**Test Group**	2T5	2	21461.12708
**Test Group**	2T6	2	6164.18508
**Test Group**	2T7	2	655.67900
**Test Group**	2T8	2	2972.12553
**Test Group**	2T9	2	46665.45516
**Test Group**	2T10	2	66798.01003
**Test Group**	2T11	2	44074.34501
**Test Group**	2T12	2	186243.95412
**Test Group**	2T13	2	1059.64068
**Test Group**	2T14	2	42712.44791
**Test Group**	2T15	2	54450.18513
**Test Group**	2T16	2	89413.94445
**Test Group**	2T17	2	5746.94219

**Test Group**	2T18	2	887715.42978
**Test Group**	2T19	2	29524.08579
**Test Group**	2T20	2	5765.82952
**Test Group**	5T1	5	12474.30081
**Test Group**	5T2	5	7722.02351
**Test Group**	5T3	5	1926185.51823
**Test Group**	5T4	5	0.00000
**Test Group**	5T5	5	873590.97981
**Test Group**	5T6	5	0.00000
**Test Group**	5T7	5	1214387.36708
**Test Group**	5T8	5	8154.98304
**Test Group**	5T9	5	0.00000
**Test Group**	5T10	5	159324.23795
**Test Group**	5T11	5	0.00000
**Test Group**	5T12	5	16387.06429
**Test Group**	5T13	5	4140775.56947
**Test Group**	5T14	5	0.00000
**Test Group**	5T15	5	10914.08110
**Test Group**	5T16	5	10929675.40593
**Test Group**	5T17	5	11785.25518
**Test Group**	5T18	5	17797487.18114
**Test Group**	5T19	5	0.00000
**Test Group**	5T20	5	25341.83029
**Test Group**	7T1	7	1347471.68228
**Test Group**	7T2	7	62538.47815
**Test Group**	7T3	7	30957768.11282
**Test Group**	7T4	7	85917714.16609
**Test Group**	7T5	7	50417318.88030
**Test Group**	7T6	7	680457.08414
**Test Group**	7T7	7	7698831.91620
**Test Group**	7T8	7	195391.90254
**Test Group**	7T9	7	0.00000
**Test Group**	7T10	7	0.00000
**Test Group**	7T11	7	0.00000
**Test Group**	7T12	7	1115.85209
**Test Group**	7T13	7	2433954.96424
**Test Group**	7T14	7	0.00000
**Test Group**	7T15	7	1012278.46702
**Test Group**	7T16	7	57839880.47384
**Test Group**	7T17	7	1406784.97924
**Test Group**	7T18	7	1031122.55023
**Test Group**	7T19	7	0.00000
**Test Group**	7T20	7	139930.19174
